# Subsoil Arbuscular Mycorrhizal Fungi for Sustainability and Climate-Smart Agriculture: A Solution Right Under Our Feet?

**DOI:** 10.3389/fmicb.2019.00744

**Published:** 2019-04-12

**Authors:** Moisés A. Sosa-Hernández, Eva F. Leifheit, Rosolino Ingraffia, Matthias C. Rillig

**Affiliations:** ^1^ Plant Ecology, Institute of Biology, Freie Universität Berlin, Berlin, Germany; ^2^ Berlin-Brandenburg Institute of Advanced Biodiversity Research (BBIB), Berlin, Germany; ^3^ Department of Agricultural, Food and Forestry Sciences, Università di Palermo, Palermo, Italy

**Keywords:** arbuscular mycorrhiza, subsoil, soil depth, agriculture, sustainability, climate-smart

## Abstract

With growing populations and climate change, assuring food and nutrition security is an increasingly challenging task. Climate-smart and sustainable agriculture, that is, conceiving agriculture to be resistant and resilient to a changing climate while keeping it viable in the long term, is probably the best solution. The role of soil biota and particularly arbuscular mycorrhizal (AM) fungi in this new agriculture is believed to be of paramount importance. However, the large nutrient pools and the microbiota of subsoils are rarely considered in the equation. Here we explore the potential contributions of subsoil AM fungi to a reduced and more efficient fertilization, carbon sequestration, and reduction of greenhouse gas emissions in agriculture. We discuss the use of crop rotations and cover cropping with deep rooting mycorrhizal plants, and low-disturbance management, as means of fostering subsoil AM communities. Finally, we suggest future research goals that would allow us to maximize these benefits.

## Introduction

Assuring food and nutrition security has long been one of the greatest challenges for humanity and given current population growth and climate change scenarios, this is an increasingly challenging task. Some of the latest estimates predict the need to increase agricultural productivity by at least 70% by 2050, and the focus shifts increasingly to the role of soil biodiversity in general (Bender et al., 2016) and particularly arbuscular mycorrhizal (AM) fungi (Thirkell et al., 2017), in achieving this in a sustainable way. Moreover, agricultural productivity needs to become more resistant and resilient to the increasingly common and severe extreme climate events, that is, agriculture needs to get climate-smart (Lipper et al., 2014).

Arbuscular mycorrhizal fungi are a monophyletic, widespread group of fungi that form a mutualistic relationship with most land plants, including many agricultural crops (Smith and Read, 2008; Brundrett and Tedersoo, 2018). While predominantly known for their ability to increase plant nutrient uptake and productivity (Smith and Smith, 2011), they influence a wide range of ecosystem processes (Rillig, 2004; Powell and Rillig, 2018). AM fungal biomass abundance (Higo et al., 2013), spore numbers (Jakobsen and Nielsen, 1983; Oehl et al., 2005; Muleta et al., 2008; Säle et al., 2015), and root colonization levels (Sutton, 1973; Jakobsen and Nielsen, 1983) typically decline with increasing soil depth, but over 50% of AM fungal total biomass can be found below 30 cm (Higo et al., 2013), and outside of agriculture, AM roots have been reported as deep as 8 m (de Araujo Pereira et al., 2018). AM fungal communities below 30 cm have also been shown to differ from those in topsoil both in spore morphology-based studies (e.g., Oehl et al., 2005; Muleta et al., 2008; Säle et al., 2015) and sequencing studies, with some phylotypes being exclusively detected in subsoil (Moll et al., 2016; Sosa-Hernández et al., 2018a). There is also growing evidence for subsoil ecological specialization in some AM fungal taxa (Sosa-Hernández et al., 2018b). Moreover, in an elevated CO_2_ experiment by Rillig and Field (2003), AM root colonization increased in subsoil (here 15–45 cm) but not in topsoil, suggesting that topsoil and deeper soil AM communities might respond differently to environmental changes. Altogether, AM fungal communities below the plow layer are often overlooked but probably highly relevant components of agroecosystems that hold opportunities for management. In this paper, we review the different potential benefits of subsoil AM for agriculture, summarize the knowledge about them, and provide suggestions for future research on this topic.

## Subsoil and Climate-Smart Agriculture

In agriculture, the term subsoil refers to the soil beneath the Ap horizon, i.e., beneath the tilled or formerly tilled horizon. Considering that tillage depth is usually 20–30 cm, the vast majority of the volume of agricultural soil can be defined as subsoil, which makes even more remarkable the comparatively scarce knowledge we have and attention we pay to it as compared to topsoil. Subsoil contributions to plant nutrition range between 10 and 80%, and are expected to increase when topsoil is dry or nutrient depleted (Kautz et al., 2013). Unsurprisingly, several studies have shown no yield increase after fertilization even in nutrient-poor soils, as nutrient availability is typically characterized in topsoil and potential nutrient delivery from subsoil was not considered (Kautz et al., 2013). Guaranteeing plant access to the subsoil nutrient and water reservoir greatly increases the resistance of the system, making a greater pool of resources available and allowing the plant to avoid detrimental conditions in the topsoil, e.g., during a drought event.

Biodiversity is assumed to stabilize ecosystem functioning under fluctuating environmental conditions, known as the insurance hypothesis (Yachi and Loreau, 1999), and Isbell et al. (2015) showed that biodiversity adds to the resistance of ecosystem productivity under climate extremes. We now also start realizing the potential impacts of soil biodiversity loss or alteration on human health (Wall et al., 2015) and food properties and quality (Rillig et al., 2018). While microbial abundances commonly decrease with increasing soil depth, subsoils can also be a microbial biodiversity reservoir and harbor unique taxa (Fierer et al., 2003), and subsoil communities have been hypothesized to contribute to the recolonization of topsoil after perturbation (An et al., 1990; Verbruggen et al., 2012), adding resilience to the system.

## Subsoil Arbuscular Mycorrhizal Fungi for Sustainable Agriculture

### General Aspects

Subsoil AM fungi communities can be abundant (Wortmann et al., 2008; Higo et al., 2013) and unique (Moll et al., 2016; Sosa-Hernández et al., 2018a) and they likely contribute to plant performance and ecosystem functioning in an underappreciated manner. In contrast with topsoil, subsoils are typically characterized by higher bulk densities and compaction, reduced pore spaces, and lower oxygen concentrations (Lynch and Wojciechowski, 2015; Weil and Brady, 2016), altogether representing a suboptimal environment for roots. Although we still lack empirical evidence of subsoil AM fungal-specific traits, it is a fair assumption that they are adapted to these environmental conditions. Among the hypothesized traits of these subsoil-specialized AM fungi would be an increased ability to colonize even the smallest soil pores, enhanced tolerance to anaerobic conditions, and, due to the general scarcity and uneven distribution of roots, greater persistence in time in the form of resting structures or long-lived mycelium. All these traits could be well-matched to the intrinsic problems a plant faces in subsoil, and could become particularly important under certain circumstances, such as present in clay soils, soils with high compaction, or soils with aeration problems. Moreover, applying a competitor-stress tolerator-ruderal (CSR) framework to AM fungi (Chagnon et al., 2015), subsoil AM fungi are expected to follow a stress tolerator life strategy. As such, deeper soil AM fungal phylotypes are expected to exhibit greater resource use efficiency and production of long-lived biomass, representing an advantageous carbon cost/benefit investment for the plant. These slow-growing communities would initially represent a carbon sink for the plant with little immediate benefits, but once the fungal network has been established, a long-lasting mycelium would provide its services to the plant at perhaps relatively little additional cost. Following the same rationale, the observed decrease in AM fungal spores with depth (e.g., Oehl et al., 2005; Muleta et al., 2008; Säle et al., 2015) might be less related to a decrease in abundance than to a change in both environment and life history strategy. AM fungal spores can be dispersed by wind (Egan et al., 2014), small mammals (Janos et al., 1995), earthworms (Reddell and Spain, 1991), or arthropods (McIlveen and Cole Jr., 1976), but all these vectors seem unlikely to be relevant in subsoils, with perhaps the exception of earthworms. With less disturbance and decreased microbial activity, probably a long-lived mycelium is in itself the best option for dispersal in time, and at larger time scales, also in space. This again represents a potential advantage for the plant symbiont, since AM fungal spores are particularly large and filled with lipids and carbohydrates with a high metabolic cost (Giovannetti, 2000), and ultimately it is the plant that provides this carbon and energy. While the same holds true for the production of mycelium, plants obtain a direct profit from this carbon investment, because it is the mycelium that explores the soil and captures and transports nutrients to the plant. Summing up, plants may receive greater returns for every unit of carbon they provide to AM fungi in subsoil, as compared to in topsoil.

**Figure 1:**
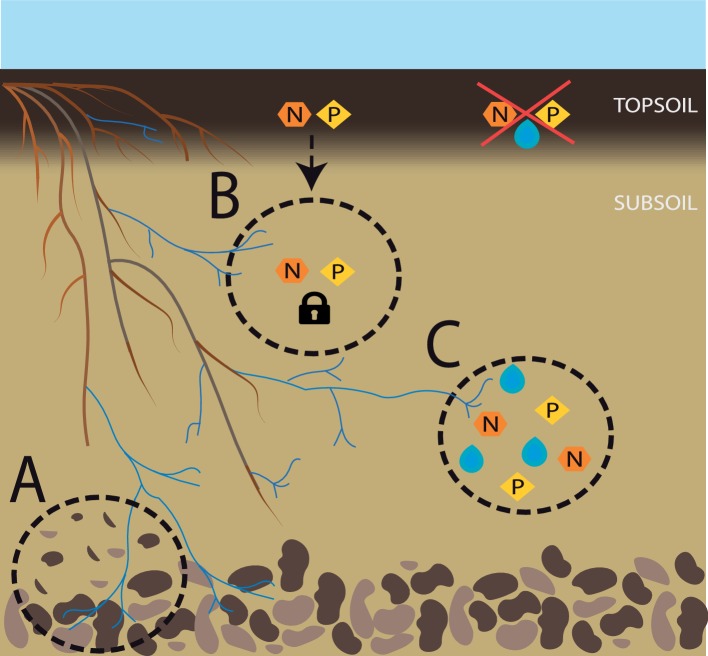
Subsoil AM fungi for sustainable agriculture. Overview of the contributions of subsoil AM fungi to a sustainable agriculture. **(A)** Enhanced soil formation; **(B)** reduction of nutrient leaching; **(C)** access to deep nutrient and water pools, particularly when suboptimal conditions prevail in the topsoil.

Last but not least, subsoil arbuscular mycorrhizae may have a significant role in the very formation of soil. The importance of the biological component in pedogenesis has long been identified (Jenny, 1994) and while bacteria tend to have greater geochemical capabilities, fungi can weather rocks too, especially mycorrhizal fungi (Hoffland et al., 2004). In fact, it is difficult to understand pedogenesis throughout earth’s history without considering the coevolution of plant roots and mycorrhizal fungi (Leake and Read, 2017). The ability of ectomycorrhizal (EM) fungi to release low-molecular weight organic chelators in soil, which enhances mineral weathering, remains to be shown in AM fungi. However, AM fungi affect mineral weathering through various indirect pathways, including increased respiration, soil stabilization, enhanced evapotranspiration and exudation (Taylor et al., 2009), and differences in the mineral weathering abilities of AM and EM roots might be less pronounced than previously assumed (Koele et al., 2014). When it comes to deeper soil layers, biological activity is generally lower and despite potential accumulation of clay minerals from upper horizons, usually, it comprises larger amounts of primary minerals, posing great potential for mineral weathering and nutrient release. AM fungi greatly expand the volume of soil under the influence of the symbiosis, often referred to as the mycorrhizosphere (Linderman, 1988), and in subsoil, this likely means fostering microbial activity in a greater volume of soil. This combined action of roots, AM fungi, and the associated microbial community has the potential to favor soil development, and in shallow soils where the parent material or the bedrock is close to the surface, this process could increase soil formation and deepening (Figure 1A).

### Efficient Fertilization

Probably the most widely appreciated contribution of AM fungi to plant performance is their ability to increase plant nutrient uptake, particularly of P (Smith and Smith, 2011). Harnessing the nutrient supply by AM fungi, the amount of applied fertilizer and the energy linked to its production can be reduced. A major issue in optimizing efficient fertilization is reducing the amount of nutrients lost to the system *via* leaching. AM fungi decrease nutrient leaching not only expanding the nutrient interception zone due to the development of a mycorrhizosphere, but also thanks to increased nutrient uptake, enhanced soil structure and fostering of the microbial community with associated nutrient immobilization (Cavagnaro et al., 2015, Figure 1B). Köhl and van der Heijden (2016) demonstrated that different AM fungal species differ in their ability to decrease nutrient leaching, highlighting the potential importance of AM fungal diversity. In fact, the observed increase in nutrient leaching in highly fertilized agroecosystems may be explained not only due to greater soil nutrient content, but also due to a typically reduced abundance and diversity of AM fungi (van der Heijden, 2010).

AM fungi have been shown to stabilize community productivity across gradients of nutrient availability, and to reduce plant tissue nutrient content variability along such gradients in a grassland (Yang et al., 2016). If transferable to agricultural systems, these effects would be crucial in achieving food and nutrition security particularly in regions where access to fertilizers might be limited or irregular. Moreover, expanding the available soil nutrient pool to deep soil further increases resistance, allowing for instance the maintenance of plant growth under drought conditions, where nutrients in topsoil might be present but not accessible (positionally unavailable) for the roots (Figure 1C). Altogether, with the continuously increasing prices of fertilizers and their predicted scarcity in a near future, making the most out of these resources is the only way forward and subsoil and subsoil AM fungi may prove important in this task.

#### Nitrogen

Nitrogen (N) applied in agricultural fields can be lost *via* leaching or in form of gaseous emissions. The influence of AM fungi on gaseous loss of N will be discussed later in this article, in the context of greenhouse gas emissions. As for leachate N, it occurs mostly in form of dissolved nitrate (NO_3_^−^), a particularly mobile form of N in soil. AM fungi promote soil aggregation (Leifheit et al., 2014) by improving soil structure and therefore increasing soil water-holding capacity. Additionally, AM fungi take up N preferentially in the form of ammonium (NH_4_^+^)_,_ reducing the pool of N available for nitrification and consequently reducing the mobility of N. In subsoil, AM fungi could intercept N that migrated down the profile and immobilize it or deliver it to the plant, thus avoiding N losses (Figure 1B). Moreover, the proportion of NH_4_^+^ to other N sources increases in subsoil (Kautz et al., 2013), increasing the potential role of subsoil AM fungi in mobilizing and delivering this N to the plant, assuring access to a previously unavailable pool and reducing the need for N fertilization (Figure 1C).

A particularly relevant role of subsoil AM fungi might be the capture and delivery to the plant of N weathered from rocks. Recently, Houlton et al. (2018) demonstrated that bedrock weathering might be a significant source of active N in various terrestrial environments. When this weathering occurs in deep soil layers, a big proportion of this N may be released to groundwater and ultimately to the sea (Houlton et al., 2018). In such scenarios, the presence of an active microbial community, together with deep soil root proliferation, is crucial to capture this N before it is lost from the system. Due to their unique ability to capture and transport nutrients from the soil directly to plant roots, including N (Smith and Smith, 2011), AM fungi are promising candidates for maximizing the benefits obtained from this previously ignored resource, both reducing the need of N input and avoiding the contamination of groundwater.

#### Phosphorus

When it comes to P, it is generally assumed that due to its low mobility in soils, leaching is of no importance and most effort has been spent on avoiding P loss and P-mediated eutrophication *via* topsoil erosion. However, we now know that excessive manuring, the existence of preferential pathways, or a sandy soil texture can lead to significant P leaching (Djodjic et al., 2004; Schoumans, 2015), with its associated economic and environmental consequences. The role of AM fungi in P uptake has been extensively researched (Smith and Smith, 2011), and they can reduce the need of heavy manuring due to increased and efficient P uptake. As for subsoils, here AM fungi can again increase water-holding capacity, reducing the risk of leaching; but these fungi can also intercept P that has migrated down the profile and deliver it to the plant (Figure 1B). Inputs of organic P in subsoil, mostly *via* roots but also with direct injection of organic matter, can remain inaccessible to the plant due to decreased decomposition and mineralization rates. The role of subsoil AM fungi may be particularly important in acquiring this otherwise unavailable P (Figure 1C). Moreover, Wang et al. (2017) found some evidence that AM fungi in subsoil might contribute more to plant P nutrition than topsoil AM fungi, under heavy P fertilization. Consequently, subsoil AM fungi have potential to be of great relevance in the avoidance of P loss, particularly in sandy soils or when the topsoil is P saturated.

#### Re-allocation of Nutrients

More generally, fostering the proliferation of roots and AM fungi in deeper soil layers expands the volume of biologically active soil, increasing nutrient mineralization and immobilization rates. Thanks to their unparalleled ability to penetrate even the smallest soil pores such as in high-density environments like subsoil, these fungi reach nutrients beyond the rhizosphere and transport them to the plant and topsoil again. This notwithstanding, no microorganism can increase the net content of nutrients in soil, with the exception of N-fixing bacteria. Therefore, even the most sustainable and efficient agricultural practices will eventually need to resupply nutrients to the soil. The same applies to subsoils: gaining access to this nutrient pool does not exempt farmers from the need to eventually replenish it. Natural migration of nutrients from topsoil to subsoil typically occurs *via* root exudates, dead roots, the action of anecic earthworms, and the deposition of nutrients dissolved in water that reach subsoil through preferential flow pathways (Kautz et al., 2013). Therefore, enhancing the formation and maintenance of biopores is crucial for a proper replenishment of the subsoil. Additionally, the presence of an extensive mycorrhizosphere with its associated exudates can foster the return of some nutrients to the subsoil. Apart from these natural processes, direct inclusion of nutrients in deeper layers, such as injection of organic matter into subsoil, should be considered. Recent studies have shown positive effects of the admixing of organic matter in subsoil on the performance of barley (Jakobs et al., 2017), but understanding the long-term effects of these on subsoil diversity and sustainability requires further research.

## Greenhouse Gas Emission in Agriculture

Modern agriculture is responsible for an estimated 12% of the global anthropogenic greenhouse gas emissions (Linquist et al., 2012). Some of these emissions are associated with fertilizer production and the use of heavy machinery, but most of them occur in the form of direct emissions from the field. The potential benefits of subsoil AM fungi in alleviating emissions related to fertilizer application were discussed in the previous section. Next, we will address the role of subsoil AM fungi in reducing the release of two important greenhouse gasses associated with agriculture: carbon dioxide (CO_2_) and nitrous oxide (N_2_O).

### Carbon Dioxide: Subsoil Arbuscular Mycorrhizal Fungi and Carbon Farming

The traditional view of very stable carbon in subsoil is questioned in recent findings. Stable subsoil carbon may be readily decomposed when fresh carbon is added. We propose that AM fungi have the potential to counteract this phenomenon due to their function in soil structure and in the capture of nutrients.

#### The Traditional View of Carbon in Subsoil

In depths of up to 3 m, the pedosphere stores more carbon (C) than the biosphere and the atmosphere combined (Jobbágy and Jackson, 2000). With increasing depth throughout the soil profile, the mean residence time of C increases, reaching up to 10,000 years (refs. 2–4 in Fontaine et al., 2007). In the past, it was generally assumed that the age of C is connected to its stability, i.e., older C is also more stable.

Indeed, we do find very stable compounds in the subsoil that have much slower turnover times than compounds in the topsoil [Spielvogel et al., 2008; Rumpel and Kögel-Knabner, 2011; Balesdent et al., 2018 (and refs. 16–19 therein)]. This could be attributed to several reasons:

(1) Subsoils usually have reduced amounts of energy sources and nutrients, especially N and P, which limit microbial activity and thus the turnover of OM. (2) Subsoils have a higher soil density with smaller pore volumes that decrease overall habitat space for soil organisms, thus reducing their abundance. (3) Subsoils often show a change in texture, i.e., increased amounts of clay that can bind organic matter (OM) in organo-mineral complexes with stable bonds resulting from, e.g., ligand exchange or polyvalent cation bridges. As environmental conditions such as temperature and moisture are usually more stable in subsoil (Weil and Brady, 2016), the importance of soil mineral chemistry for OM stabilization becomes more pronounced. (4) In subsoil, a greater proportion of OM is located in microaggregates as compared to topsoil, allowing for slower turnover times (Torres-Sallan et al., 2017).

#### Recent Findings Question the Stability of C in Subsoil

However, in more recent studies, the stability of old C in the subsoil has been questioned and a number of studies have shown that subsoil C is susceptible to decomposition when fresh C is added to the soil (e.g., Fontaine et al., 2007; Hobley et al., 2017). The majority of these studies extracted the soil for use in pot studies, where single and sometimes easily degradable substances were added to the soil. The soil extraction represents a massive disturbance, changes temperature, soil density, and moisture conditions, which strongly boost microbial activity and thus degradation of OM (Rumpel and Kögel-Knabner, 2011). Therefore, the instability of subsoil OM might have been overestimated due to methodological flaws and could be much less in the field under realistic conditions.

#### The Role of Arbuscular Mycorrhizal Fungi in Subsoil Carbon Cycling–Soil Structure

**Figure 2:**
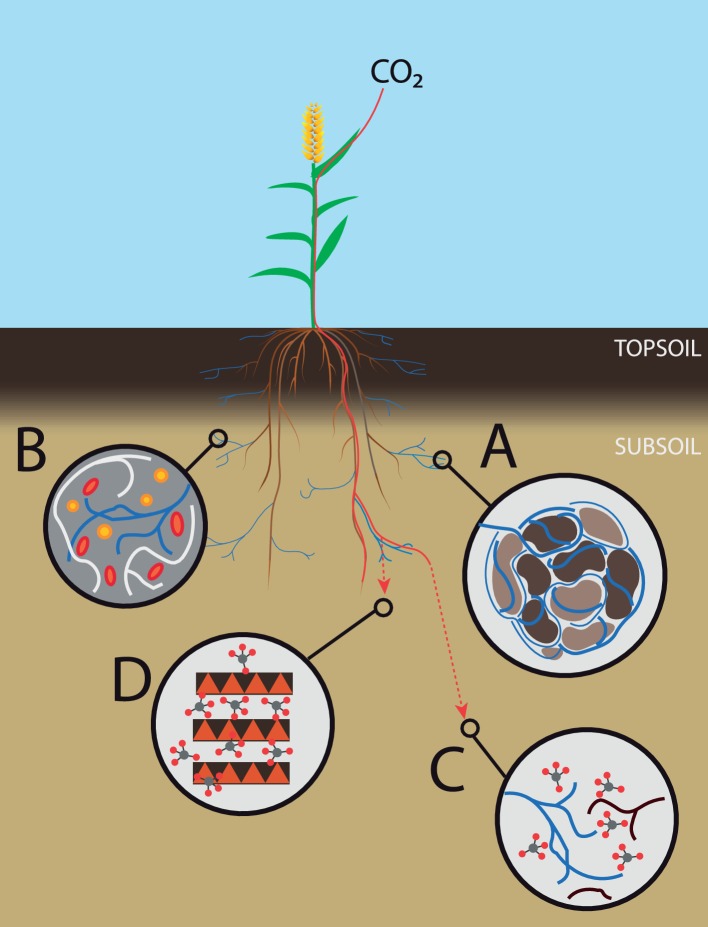
Subsoil AM fungi and carbon farming. Overview of different benefits of subsoil AM fungi on carbon sequestration. **(A)** Improvement of the soil structure, leading to aggregate-protected organic matter. **(B)** Competition with saprotrophic bacteria and fungi, thus reducing decomposition rates. **(C)** Increased carbon input in subsoil *via* mycelial exudates and turnover. **(D)** Formation of highly stable mineral-associated organic matter fractions.

One factor usually not included in previous experiments considering subsoil C cycling is AM fungi. In numerous studies, they have been shown to improve soil aggregate stability through hyphal enmeshment of soil aggregates and the production of extracellular polymers (Rillig and Mummey, 2006). Compared to topsoil, subsoil is subject to less disturbance that can disrupt hyphal networks, leading to a longer residence time of aggregate-protected OM (Lehmann et al., 2017). Therefore, stabilization of soil aggregates by mycorrhizal hyphae in the subsoil can contribute substantially to the protection and thus sequestration of soil organic matter (SOC) (Figure 2A). A better soil structure also improves soil pore connectivity, leading to increased interactions between soil microbes, and, consequently, likely increased competition for nutrients. If AM fungi could outcompete decomposers for nutrients, they would be able to indirectly reduce decomposition activity and thus potential loss of added or stabilized carbon (Figure 2B).

#### The Role of Arbuscular Mycorrhizal Fungi in Subsoil Carbon Cycling–Nutrient Additions

More specifically, competition for nutrients can also be induced directly by AM fungi, as they acquire nutrients and water for themselves, thereby reducing the nutrient and water availability for other microbes, which could reduce the activity of decomposers due to nutrient or water deficiency (Verbruggen et al., 2013; Jansa and Treseder, 2017, Figure 2B). However, nutrient additions, as single or combined additions of N and P increase SOC decomposition, an effect called priming (Kuzyakov, 2010). Meyer et al. concluded that both the current soil nutrient conditions and microbial nutrient demand must be considered when predicting the effect of N addition on SOC turnover. According to the authors, the importance of the subsoil as a long-term C sink is unclear when there is also increased input of additional N and P. The nutrient capture by AM fungi could be important for minimizing the stimulating effect that additional nutrients have on decomposition of SOM, particularly after the admixing of organic matter in subsoil, as performed by Jakobs et al. (2017).

In addition to capturing nutrients, AM fungi can reduce the availability of carbon compounds in the rhizosphere, because plants provide carbon to AM fungi in exchange for nutrients delivered (Jones et al., 2004). In the absence of AM, higher rhizodeposition would stimulate microorganisms in the rhizosphere, and thereby possibly stimulate SOM decomposition as microorganisms mine for nutrients in stabilized SOM. AM fungi receive up to 20% of a plants’ assimilates (Bago et al., 2000), which they first use for their own metabolism, before mycelial exudates are released. In this way, the mycorrhizal extraradical mycelium can be an important pathway of C to the SOM pool, when they exude mycelial organic compounds to soil parts more distant from the root system, but also *via* mycelium turnover (Figure 2C). In topsoil, the C input by mycorrhiza can sometimes exceed the input of leaf litter and fine root turnover. In a boreal forest, Clemmensen et al. (2013) found that in subsoil, up to 70% of soil C can be root-derived, especially when root densities were high in deep horizons. In this study, and several others, mycorrhizal and other endophytic fungi dominated the subsoil, but decomposer fungi were only abundant in upper soil horizons. This suggests that decomposition processes controlled by microbial community composition *in situ* might be dominant in topsoil but subordinate in subsoil.

#### The Role of Arbuscular Mycorrhizal Fungi in Subsoil Carbon Cycling–Litter Decomposition

Although AM fungi may increase litter decomposition in short-term laboratory experiments, they probably have positive long-term effects on soil C. In the short term, AM fungi are able to enhance OM degradation through the stimulation of decomposers, but we do not know whether this stimulation is permanent. Moreover, microbial metabolites are not necessarily lost, they can be integrated into very stable compounds such as mineral-associated SOM fractions, which have the longest mean residence times in soil (Figure 2D). Indeed, subsoil OM contains more microbial-derived compounds compared to topsoil and microbially processed sugars seem to better associate with the mineral phase than plant-derived OM (Rumpel and Kögel-Knabner, 2011). This stabilization mechanism could be especially important in subsoil, because here, the amount of clay minerals and sesquioxides increases, representing a great potential for long-term stabilization of (fresh) C.

#### The Potential Contribution of Arbuscular Mycorrhizal Fungi in Subsoil Carbon Storage

Some efforts are made to find ways to increase SOC storage, e.g., in subsoil by increasing the presence of plants throughout the year with catch crops, by the use of undersown crops or deep rooting plants (Kell, 2011; Jakobs et al., 2017). Without further management, however, this could stimulate soil microbial activity and thus also decomposition of freshly added OM as well as stabilized OM (Kong, 2018). To counteract this effect, AM fungi could be fostered in order to reduce rhizodeposits, by including mycorrhizal crops together with a low management intensity (e.g., no tillage) and adapting a low fertilization level, as mycorrhizal fungi are more abundant in no-tillage systems and their effects are more pronounced in nutrient-limited systems (Jansa et al., 2002, 2006). However, the interaction of plants, AM fungi, and other microbes in relation to SOC storage in soil particles or microbial biomass is still not very well understood. For instance, although AM fungi have been observed to induce smaller priming effects on SOM than roots, they might still promote soil respiration and thus increase SOC losses. Therefore, future research should adopt a comprehensive approach for studying plant—fungal-mediated processes in C cycling, considering the influxes (e.g., photosynthetic assimilation, root exudation, mycelial exudation, litter fall, soil organism detritus and fecal residues), effluxes (e.g., all parts of soil respiration, decomposition, leaching), as well as immobilization and storage of C in SOM and microbial biomass. These processes are especially interesting to study with respect to long-term C gains, e.g., through plant growth promotion effects, soil aggregation, or the production of microbial products.

Data on the sensitivity of stored deep C are limited; we need further on-site research (with a low level of disturbance and alteration of environmental conditions) to evaluate the impact and importance of management strategies such as deep rooting plants, and effects of microbial community properties.

### Nitrous Oxide Emissions

**Figure 3:**
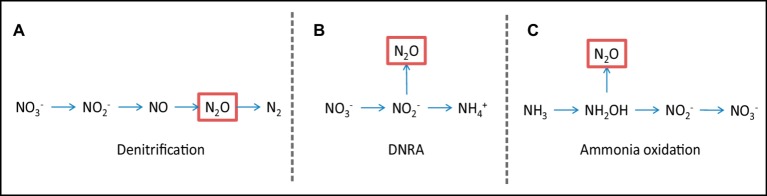
Nitrous oxide emissions. Simplified overview of N_2_O (nitrous oxide)-producing processes that can be influenced by AM fungi. **(A)** Denitrification, **(B)** dissimilatory nitrate reduction to ammonium (DNRA), **(C)** ammonia oxidation.

Agriculture is a major source of anthropogenic N_2_O emissions (Linquist et al., 2012), a potent greenhouse gas with tremendous global warming potential 280–310 higher than CO_2_ and a lifetime in the atmosphere that ranges from 118 to 131 years (IPCC, 2001; Fleming et al., 2011). Multiple pathways of N_2_O production co-occur in soil and their relative contribution to its emission is poorly understood. Ammonia oxidation, dissimilatory nitrate reduction to ammonium (DNRA), and various denitrification pathways have been identified as microbially mediated processes with significant contributions to N_2_O emission in agricultural soils (Baggs, 2011; Zhu et al., 2013, Figure 3). Under low oxygen concentrations, such as those expected in subsoil, typically anaerobic processes, such as denitrification or DNRA (Figures 3A,B), are expected to prevail (Baggs, 2011), with significant denitrification rates having been reported in subsoil (Cleemput, 1998; Clough et al., 2005). Since NO_3_^−^ is the primary substrate for both processes, we can expect that the reduction in NO_3_^−^ leachate arriving at the subsoil due to the effect of AM fungi would also have a negative impact on DNRA and denitrification rates in subsoil. Furthermore, in grassland subsoil, the addition of easily available C increased N_2_O production, suggesting again that the reduced secretion of simple carbohydrate exudates in an AM root would further reduce this process. Ammonia oxidation is an aerobic process mediated by autotrophic organisms, in which the concentrations of oxygen and the substrate ammonia (NH_3_) influence process rates (Figure 3C). AM fungi were shown to have a direct negative effect on N_2_O emission following N fertilization in a pot trial using agricultural soil, and the competition with nitrifiers for NH_4_^−^ was identified as the main driver (Storer et al., 2018). While the presence of high NH_4_^−^ concentrations in subsoil is unlikely due to its limited mobility, this might not be the case following the mineralization of admixed organic matter in subsoil. Under such scenarios, where additionally considerably less anaerobic conditions prevail due to the deep tillage, the presence of subsoil AM fungi to readily take NH_4_^−^ up and outcompete nitrifiers would be potentially important.

## Potentials and Limitations to Promote Beneficial Effects of Arbuscular Mycorrhizal Fungi in the Subsoil by Agricultural Management

Achieving food security at a global scale is a complex task requiring multiple approaches. As for increasing and securing agricultural productivity, climate-smart agriculture offers the best perspectives for success (Lipper et al., 2014). Much more research is needed to fully understand the role of subsoil and subsoil AM fungi in plant performance and to what extent we can manage them for sustainable intensification. This notwithstanding, evidence begins to accumulate pointing at particular agricultural practices that may help make our yields more sustainable and climate-smart (Table 1). First and foremost, we need to approach these challenges in a well-informed and integrated way, as optimizing only some aspects of productivity while ignoring others will certainly be counterproductive (Rillig et al., 2016). In fact, there is no one-size-fits-all solution and required management components are highly context dependent. This is why sustainable intensification has been defined as an increase of knowledge per hectare (Buckwell et al., 2014), stressing the importance of fine-tuned information.

**Table 1:** Suggested management approaches to foster subsoil AM fungi.

Management	Aim
Crop rotation	Including deep rooting and mycorrhizal plants in crop rotations to increase deep soil root proliferation and AM abundance
Catch crops and cover crops	Catch crops and cover crops can increase AM abundance through the profile, increasing AM colonization for the next crop
Crop breeding and selection	Plant breeding and selection of crops with a focus on mycorrhizal responsiveness and deep rooting traits
Reduced/no till	Reduced and no-till systems typically increase AM abundance
Deep plowing	In the presence of a plow pan that restricts root growth into subsoil, deep plowing can allow for subsoil root and AM fungal proliferation

### Plant Breeding and Choice

Clearly, a fundamental prerequisite for the exploitation of subsoil is the presence of deep roots. Thus, crop rotation or catch cropping with deep rooting plants is essential to access deep soil resources and to create biopores that subsequent crops can use to grow into subsoil (Kautz et al., 2013). For instance, deep rooting and mycorrhizal plants, such as wheat, have been shown to increase AM fungal abundance through the soil profile (Higo et al., 2013). The use of cover crops has also been identified as a means of increasing AM fungal inoculum in soil (e.g., Galvez et al., 1995; Boswell et al., 1998; White and Weil, 2010; Lehman et al., 2012). Additionally, crop breeding and crop selection can be done considering a set of traits that favor the plant’s abilities to access subsoil, as reviewed by Bishopp and Lynch (2015) and Lynch and Wojciechowski (2015). However, it is very unlikely that any one given cultivar will possess all the traits required to fully optimize the use of subsoil. Therefore, while developing crop rotations or intercropping systems, it is desirable to look closely at the roots and select for a varied and balanced set of traits that better suits our goals (Rillig et al., 2015), aiming not only for a diversity of aboveground characteristics but a diversity of root architectures and abilities that can sustain the desired ecosystem services (Bardgett et al., 2014; Bardgett and van der Putten, 2014). Plant domestication has produced high-yielding and resistant phenotypes that perform better than their wild relatives in the context of high-input agriculture. This selective breeding has often come at a cost of neglected impacts on the soil microbiome (Pérez-Jaramillo et al., 2016). In the particular case of AM fungi, an extensive analysis comparing domesticated plants with their wild relatives found that under limited P availability, both phenotypes profit from AM colonization, but under high P fertilization regimes (such as in conventional agriculture), the symbiosis was less efficient in domesticated plants (Martín-Robles et al., 2018). In addition to deep rooting traits, we recommend accounting for mycorrhizal responsiveness in future plant breeding efforts to assure that crops can benefit the most from the local AM fungal communities (Rillig et al., 2016).

### Subsoil Management

Access to subsoil can be limited by physical properties, such as the existence of a hard plow pan that prevents root growth. The benefits of deep tillage and other subsoil tillage management options can be controversial and highly context dependent; but on average, given the existence of a plow pan, yields can be substantially increased after deep plowing (Schneider et al., 2017). The existence of subsoil-specific AM fungal phylotypes and their inability to survive soil mixing events, however, calls for precaution and the general avoidance of any method that inverts the soil profile (Sosa-Hernández et al., 2018b). Intensive tillage has been identified as a major factor reducing AM fungal abundance and diversity in agriculture (Kabir, 2005). Recently, Säle et al. (2015) compared the effects of reduced and conventional tillage, down to 40 cm in the soil profile using spore-based community analysis. Their results confirm the expected shifts in spore abundance and diversity in topsoil but those effects were not significant in deeper layers, despite a shift in community composition. The absence of spore abundance shifts does not necessarily imply a lack of effect on hyphal abundance or colonization rates, but changes in subsoil community composition highlight that tillage can affect AM fungi in deeper layers, with unknown consequences for their functionality. No-till or reduced till systems however typically face another set of problems that may include increases in bulk soil density, limited nutrient mobility through the profile, or the use of agrochemicals for weed control, plus a set of economic and technical constraints that are more pronounced on small farms (Giller et al., 2015).

### Arbuscular Mycorrhizal Fungal Inoculum

Assembling the right consortia of plant phenotype and rhizosphere microbiome has also been postulated as one of the means for a new underground revolution that aims at an ecological intensification in agriculture (Bender et al., 2016). This approach is very promising but holds intrinsic associated risks (Machado et al., 2017). The benefits of mycorrhizal inoculum can be highly context dependent (Hoeksema et al., 2010) and the use of non-native genotypes carries always the possibility of associated environmental impacts (Schwartz et al., 2006). This variability (but often not uncertainty, (Lehmann and Rillig, 2014)) in response to AM inoculation often leads to a lack of trust in its general efficiency by the agricultural community. We think AM fungal inoculum should not be used indiscriminately in general, or substitute for other AM-promoting management options. When it comes to subsoils, the evident existence of a specific AM fungal community calls for even greater caution, and at present, our knowledge is too limited to encourage the use of inoculum for the subsoil.

## Future Research Challenges

Early research on AM fungi already observed abrupt decreases of spore abundance and colonization levels with increasing depth in agriculture (Sutton and Barron, 1972; Sutton, 1973). This could have led to a subsequent lack of interest in studying the arbuscular mycorrhizal symbiosis in deeper layers. However, outside the realm of agriculture, evidence of AM colonization was found down to 4.8 m in honey mesquite (Virginia et al., 1986) and this depth record has been recently updated to 8 m in an *Eucalyptus* and *Acacia* plantation (de Araujo Pereira et al., 2018). Very little research has been conducted on the community composition of AM fungi across different depths in agriculture, with few notable exceptions (e.g., Oehl et al., 2005; Muleta et al., 2008; Säle et al., 2015), and these spore-based studies have only recently been supported by molecular-based research (Moll et al., 2016; Wang et al., 2017; Sosa-Hernández et al., 2018a). Moreover, the only assessment on subsoil AM functionality was performed by Hafner et al. (2014), who compared root-derived C in the rhizosphere as influenced by AM fungi from two different depths in a greenhouse experiment.

Consequently, we believe that more basic, descriptive research, both spore and molecular based, needs to be performed to better understand the vertical distribution of AM fungi in agriculture and to confirm some of the already obtained knowledge across different regions and crops. We think it is particularly important to start linking agricultural management with responses in AM fungi across the entire soil profile, as exemplified by Säle et al. (2015), ideally covering aspects such as tillage, fertilization, and crop rotations. Furthermore, we also need to learn about the functioning of AM fungal communities in the subsoil, since AM fungi and roots face a very different environment than in topsoil. Rooting depth and architecture is one of the niche axes that allows plant coexistence in natural habitats (Silvertown, 2004), and roots at varying depths may forage for different resources (e. g., shallow roots acquiring P and deeper roots acquiring water). We can assume that, similarly, what the plant demands from its mycorrhizal partner might vary with soil depth, opening the possibility for specialized or even new functionality of subsoil AM phylotypes. Experiments assessing these potential differences in mycorrhizal functionality across depths are crucial and the isolation of deep soil AM fungi would go a long way toward the understanding of these communities.

Discerning the assemblage mechanisms, ecosystem role, and phylogenetic structure of AM fungi in deeper soil layers will help us answer important questions about AM fungal biogeography and diversity maintenance. Despite the three-dimensional nature of soil, to date, we have centered most of our efforts on a shallow soil layer with virtually no understanding of the ecosystem contributions of deeper AM fungi (Powell and Rillig, 2018), even if most evidence points to greater vertical than horizontal variation in fungal community composition (Bahram et al., 2015). Routinely including the vertical axis in AM studies across different biomes and in our theoretical frameworks will deepen our overall understanding of the biology of this relevant group of plant symbionts. Increasing our knowledge and expanding our perspective to include subsoil and subsoil AM fungal communities will not solve our problems on its own; however, an integrated subsoil management that takes AM fungi into account can bring us one step further in achieving sustainable and stable yields.

## Author Contributions

MS-H wrote the first draft of the paper; EL, RI, and MR contributed ideas and text.

### Conflict of Interest Statement

The authors declare that the research was conducted in the absence of any commercial or financial relationships that could be construed as a potential conflict of interest.
